# Woman’s Part in Helping to Enforce the Pure Food Laws

**Published:** 1912-08

**Authors:** 


### Woman's Part in Helping to Enforce the Pure Food Laws.

When I remarked to some friends of mine that I would have an article from the Pure Food Department in this number of the JOURNAL, they laughed, and asked me if I considered the question of Pure Food vital.

Well, yes; I consider it one of the most vital things in life; and there are some people who think that one's happiness and success depend very greatly on what is put in the stomach.

Some of our National and State leaders thought this a question of grave importance, and after years of struggle with the corporations and trusts and patent medicine combines they succeeded in establishing, in 1906, a department for the protection of our foods.

This law would have afforded us complete protection from adulterated, misbranded, and harmful foods, drugs, liquors and medicines had Dr. Wiley been permitted to carry out the spirit and letter of the law. The men and women who believe in "equal rights for all, special privileges to none," should not forget that President Taft has retained in office Secretary Wilson, Messrs. McCabe and Dunlap, when these men have steadily upheld the interest of the men who deemed the common people their prey. Dr. Wiley is a good, conscientious man who, rather than have his hands tied by these "sleuth-hounds of greed," resigned; and every man and woman in the United States should demand the expulsion from office of these men who have made it impossible for Dr. Wiley to execute the law.

Now, I do not subscribe to the Epicurean doctrine—"Eat, drink and be merry; tomorrow ye die." I should like to paraphrase this ancient and oft-quoted expression, and have it read like this:

Eat—sparingly—and of pure food.
Drink—abundantly—of pure water.
Be cheerful—and you will live to a ripe old age.

It has been said, and truly, I believe, that "Americans dig their graves with their teeth." What we want is quality and not quantity.

Believing that every woman should know something about the adulteration of our simplest foods, I asked Dr. Abbott to write an article on this subject for the JOURNAL, and he has done so. It appears herewith.

Dr. Abbott has spent years in the study of food values, and he is anxious that every wide-awake woman will co-operate with him in this great work. The Pure Food Commission was established to put the men who are selling adulterated stuff out of business, and Dr. Abbott and his able staff are surely doing their share; but there must be co-operation on the part of the housekeepers. They must demand pure food.

The groceryman is not wholly to blame that you use adulterated stuff. Many of them are quite as honest as little Mary, who on being asked by a customer—who had just finished his third glass of lemonade—why she sold her lemonade cheaper than her little friend at the next booth, she replied, "Well, I'll tell you, if you won't mention it to anyone else. The puppy fell in mine, and I honestly thought I should sell it a little cheaper than the others."

The groceryman will tell you that a certain brand of tomatoes is cheaper because there is no "red paint" in them, and they do not appear so attractive when opened. They do not try to keep these things a secret. The reason we do not know is because we have not taken time to ask. The honest groceryman will be delighted to have you examine his goods and he will take more pride in keeping things clean if he knows that you expect him to do so. Men are not innately good housekeepers, but they would be more cleanly if they knew the trade of a customer depended upon it.

Dr. Abbott asks: "Is it not perfectly reasonable to say that the housewife should spend a little time studying some of our common articles of diet?"

It seems that she would be only too glad to study these questions, seeing they often jeopardize the lives of those for whom she is responsible. It is only a matter of time, I think, when the women's clubs will take up this matter.

But why wait until the clubs popularize the subject? Why not form little "Pure Food Clubs" in every neighborhood, and once in awhile get together and discuss these problems? Let each one preserve whatever she may find in her reading, and present it to be read aloud in the club. Dr. Abbott's article would be fine for the first meeting. If you think you are just a little more advanced in your way of thinking, and if you really want to help your neighbor, why not try this? I will be glad to send you some literature on the subject if you wish to organize a club.

JOSEPHINE DANIEL.

SOME OF THE SUBJECTS THAT WILL BE DISCUSSED IN THE "RED BACK."

Woman's Part in Helping to Enforce the Pure Food Laws.
The Public Drinking Cup—A Menace to Civilization.
Ignorance the Cause of Blindness.
The Influence of Pictures Upon a Child.
The Emancipation of Woman.
How to Tell a Child the Story of Life.
Qualifications for Parenthood.
School Hygiene.
The Power of Ideals.
Efficient Motherhood.
Shall We Have One Standard of Morals?—What Shall Be the New Standard?
What Parents Should Teach Their Children.
The Right of Babies to be Well Born.
Medical Inspection in Schools.

---

DALLAS, TEXAS, July 1, 1912.

*Mrs. Josephine Daniel, 204 East Ninth Street, Austin, Texas.*

DEAR MRS. DANIEL: It will give me great pleasure to contribute towards making successful any efforts that you may put forth in connection with a department of special interest to women. I think that we can now foresee the day when woman will come into her own and will be regarded as a helpmeet indeed, sharing fully with her husband in all things temporal. I can see no reason whatever for any broad-minded man feeling that he is doing an undignified thing by giving approval of any movement that would tend to promote the interests of our sisters.

I shall be only too glad to aid you in anyway that I can, and I can not too strongly urge the other wide-awake and up-to-date doctors of Texas to give you such assistance in your laudable undertaking as it is possible for them to do; for unquestionably this is a good movement in my judgment.

Wishing you every success with your new department, and assuring you of my hearty co-operation, I am,

Very truly ours,

JNO. S. TURNER, M. D.,
President Texas State Medical Association.

DALLAS, TEXAS, June 26, 1912.

*Mrs. F. E. Daniel, Austin, Texas.*

MY DEAR MRS. DANIEL: If all women were taught to know themselves as they should and would govern themselves accordingly, the benefits resulting would be almost beyond comprehension.

The department you are establishing in the "Red Back" along this line, giving women good, wholesome knowledge without the taint of quackery is a most worthy undertaking and deserves hearty support.

Your friend,

W. R. BLAILOCK, M. D.

---

PARIS, ILLINOIS, July 12, 1912.

*Mrs. Josephine Daniel, Austin, Texas.*

DEAR MRS. DANIEL: It was with feelings of the deepest gratification that I received your letter with the request that I should be counted as a collaborator in the new departure for the "Red Back." You may count on me to the last in this laudable work which you have undertaken, and which, I am firmly convinced, will be of the greatest benefit, as it will reach those who are most vitally concerned in the wonderful work of the emancipation of your sex from the thraldom of the old and savage idea of proprietorship, that is in too many instances a slavery more galling and degrading than that of mere chattel slavery, the more so that the victims are of the cleanest, sweetest souls of our time. This work means the removal of the curse of man's sin from the shoulders of womankind.

Thanking you for the opportunity and assuring you that I shall co-operate to the best of my limited ability, I am,

Yours respectfully,

DR. G. HENRI BOGART.

---

### THE HOUSEFLY.

Do you know where the innocent little housefly hatches? Do you know what he was before he hatched? Do you know that *one* little housefly can start an epidemic of typhoid fever?

Would you know how to exterminate the fly? If so, send your name and address to the State Board of Health at Austin, and they will send you, free of charge, a circular which will give directions for exterminating the deadly fly.

Will it cost something to exterminate the fly? Yes, it will cost you a few cents, perhaps, and a little trouble; but it is easier than sitting up nights with the sick, and cheaper than a doctor's bill or a funeral.

---

Sex Hygiene.—After months of argument, the Board of Education of Orange, N. J., has come to the conclusion that sex hygiene should be taught in the public schools. By a unanimous vote on June 11th the Board decided that girls of 14 years of age should receive instruction from competent teachers. Some effort was made along these lines several months ago, but so many protests were entered that a special committee was appointed to report on the propriety of the subject as a part of the school curriculum.

---

We are but slaves close fettered by convention;
To code and calendar we bow the knee;
Oft we proclaim our firm and fixed intention
To rise in wrath, to conquer and be free.
But when the hour arrives,
We cast away our arms and scuttle for our lives.

H. S. Baketel, M. D.

---

Dr. Philip Zenner was refused a hearing when he proposed to address one of the leading woman's clubs of Cincinnati on the subject of "Social Disease—Woman's Problem." Listen to this from far away Texas:

"Pleading upon a common sense basis for the single standard of purity for men and women, Dr. Winfield Scott Hall, of Chicago, the expert on social hygiene brought to Dallas under the auspices of the Young Men's Christian Association and the men's committee of one hundred, addressed about five hundred men at the Y. M. C. A. building.

"Later in the afternoon he spoke to about one hundred women at the First Methodist Church upon 'Sexual Hygiene,' especially considering it from the standpoint of the mother and her care of her children. At night he spoke to a large audience of young women who must meet men in business relations, at the Central Presbyterian Church. Here he dwelt upon the necessity for a proper attitude of decorum and tact upon the part of the young woman engaged in business.

"Three other short talks were made during the day, all of them to boys and young men."

And these lectures were kept up for three days at the rate of eight lectures a day. And the daily press used up columns of space in reporting the lectures. Evidently Texas is not afraid to hear of or talk of subjects of vital interest to its people.—*Lancet-Clinic,* Cincinnati.

